# Rats in Epilepsy Research: A Bibliometric Analysis of Citations Between 1969 and 2020 on Experimental Models in Epilepsy

**DOI:** 10.7759/cureus.48891

**Published:** 2023-11-16

**Authors:** Carmen Rubio, Fernando Gatica, Alonso Portila, David Vázquez, José Molina-García, Ernesto Piñón, Moisés Rubio-Osornio

**Affiliations:** 1 Neurophysiology, Instituto Nacional de Neurología y Neurocirugía, Ciudad de México, MEX; 2 Neurochemistry, Instituto Nacional de Neurología y Neurocirugía, Ciudad de México, MEX

**Keywords:** rat, bibliometric analysis, experimental model, seizure, epilepsy

## Abstract

Epilepsy stands as a prominent neurological disorder, affecting a substantial number of individuals who, unfortunately, do not respond to conventional antiepileptic medications. To unravel the intricate mechanisms underlying epileptic seizures and explore potential therapeutic avenues, researchers have turned to animal models. Among these models, rats have emerged as one of the cornerstones of epilepsy research. This bibliometric analysis embarks on the crucial task of delving into the role of rat models in deciphering the mysteries of epileptic seizures and, notably, pinpointing the most prevalent models in use.

Our study harnessed Scopus' citation tracking feature to review a range of research papers dating from 1969 to 2020, all dedicated to the exploration of epileptic seizures in rats. The citations that emerged from this rigorous process were subjected to thematic coding, primarily centered around the specific epileptic animal models employed, and subsequently, comprehensive descriptive statistics were computed.

In this effort, we found a total of 1,318 publications that explore the world of rat studies, accumulating a substantial citation count of 44,824 references. This analysis illuminated the invaluable role that research employing rat models has played in shaping our current clinical understanding of epileptic seizures. Notably, several models have emerged as predominant forces in this field, including those induced by pilocarpine, pentylenetetrazole (PTZ), kainic acid (KA), electric kindling, and electroshock.

This bibliometric exploration serves as a resounding reminder of the pivotal position that rat models occupy in advancing our comprehension of epilepsy. These findings resonate strongly, underscoring the continued importance of directing research and development funding toward this debilitating disorder, with the ultimate aim of maximizing the benefits for the patients grappling with this condition. The potential to revolutionize our approach to epilepsy and enhance the quality of life for those affected remains a beacon of hope, illuminated by the contributions of these tireless researchers and their trusty rat companions.

## Introduction and background

Bibliometric studies allow a systematic analysis of the scientific production of a certain topic of importance somewhere or in any institution at a certain time [1]. Studying the information given by the available bibliographies, the types of citations, and the relationship between these demonstrates the possible opportunities for scientific growth in this field. In fact, epilepsy is a disease that has become very important in recent bibliometric studies [2]. According to the World Health Organization (WHO), around 50 million people worldwide of all ages and genders suffer from epilepsy, of whom approximately 80% live in low- and middle-income countries. The selection of the appropriate antiepileptic drug could achieve remission of seizures in up to 70% of patients. Notwithstanding, epileptic seizure etiology is not completely understood. Evidence suggests that there are numerous mechanisms, among which are gamma-aminobutyric acid (GABA) and glutamate dysregulation. Seizures, for which no identifiable underlying cause can be determined despite the investigation of candidate genes, are referred to as idiopathic or cryptogenic. On the other hand, there is evidence suggesting a potential connection between seizures and the inflammatory process [3].

The first articles on formal experimental models of epilepsy in rats date back to 1941 with models of audiogenic seizures, such as “The effect of metrazol on the susceptibility of rats to sound-induced seizures" [4], “The latency of audiogenic seizures” [5], or “Quantitative analysis of the pattern of activity in audio-epileptic seizures in rats” [6]. However, it was not until the antiepileptic activity of valproic acid was demonstrated in the 1960s that interest in testing new antiepileptic agents in experimental models grew [7]. Animal models of epilepsy have a wide spectrum of types.

There are chemical models in which a convulsive substance is administered (penicillin, cobalt, zinc, 4 aminopyridine (4-AP), 3 mercaptopropionic acid (3-MPA), kainic acid (KA), and pentylenetetrazol (PTZ). There are also electrical models such as electroshock, ear clips, and kindling, based on brain electrical stimulation by electrodes placed in the corneas or ears, otherwise implanted in the cerebral amygdala. Finally, there are genetic models, which are used in a smaller proportion. Each model is specialized to simulate a type of seizure: focal onset (aware or impaired awareness) or generalized onset; or to simulate a specific type of epilepsy syndrome, such as the genetic absence epilepsy rats from Strasbourg (GAERS) [8, 9]. The contribution of experimental models to epilepsy research is still insufficient to fully understand the pathways and progression of seizure pathogenesis due to the limitations of using only one type of model. Therefore, to overcome these limitations, combinations of different animal models are proposed. Although most research was done on rodents, especially rats, some experiments occurred on other animals such as primates [10], cats, or rabbits [11]. Our purpose is to analyze scientific productivity and thus further elucidate how rats helped epilepsy research in a critical period of significant advances in anti-epileptic drug research.

## Review

Materials and methods

The search for all the original articles was carried out, and the main objective of the study was the application of experimental models of epilepsy in the Scopus database during the period 1969-2020. For this, our inclusion criteria were the keywords "epilepsy" or "seizure" for the title, "experimental model" and "rat". We included all articles that used either chemical, electrical, or genetic models to induce epileptic seizures in rats. We excluded all non-experimental articles as well as studies in humans or other animal species. From this database, the articles were divided depending on the model used in the experiment, according to their title, abstract, or keywords. The first classification was used to determine whether the model was physical, chemical, or genetic. Later, the type of model used was specified. If the model consisted of the administration of some substance (PTZ, KA, pilocarpine, penicillin, etc.) that was classified as chemical, on the other hand, the models of electric kindling, electroshock, hyperthermia, or surgery to induce a model of hypoxia were classified as physical. In addition, we include the following genetic models: GAERS, Wistar Albino Glaxo Rijswijk (WAG/Rij) rats, genetically epilepsy-prone rats (GEPR), as well as other types of genetic audiogenic seizures (AGS). Then, we proceeded to analyze the following study variables: citations, year, language, country, and subject area. Finally, we proceeded to analyze the articles with greater scientific impact throughout all the years studied. We also reviewed the journals that have included these articles and their impact factors, respectively.

Results

**Figure 1:**
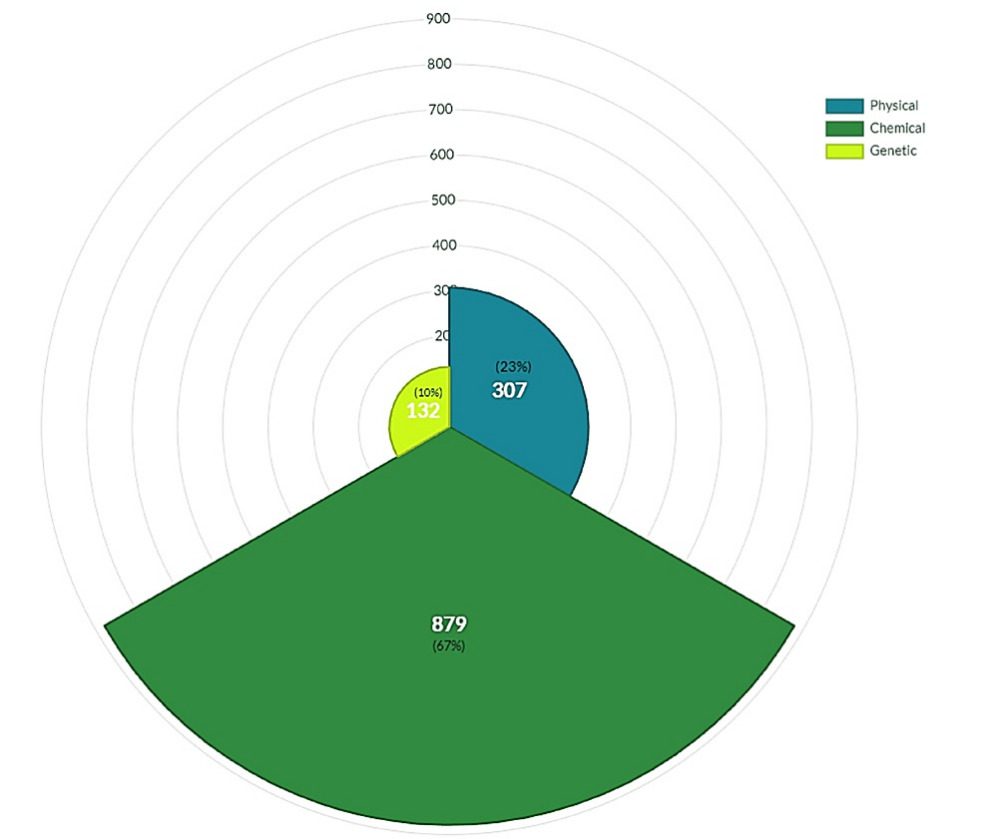
Citations of experimental models by type Of the 1318 articles, 879 belong to chemical methods, corresponding to 67%, 307 to physical methods (23%), and 132 to genetic models (10%).

A total of 1,318 articles were published during the study period (1969-2020), as found in the Scopus database. In all the articles studied, some experimental models of epilepsy in rats were used. Most methods were chemical (66.69%), followed by physical (23.29%) and genetic (10.02%) (Figure 1).

**Figure 2:**
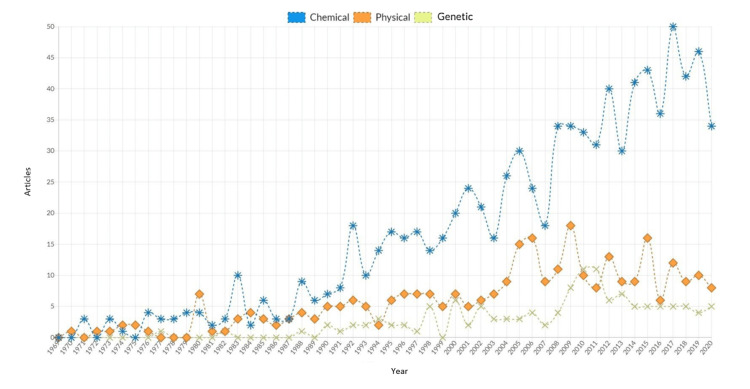
Articles published per year according to the type of experimental model between 1969 and 2020 There is an upward trend in chemical models with a boom in 1992, reaching its highest point in 2017, followed by physical models over time. Genetic models almost fell into disuse, without growth, at least in the last six years.

The distribution of these three categories of experimental models over time is shown in Figure 2.

**Figure 3:**
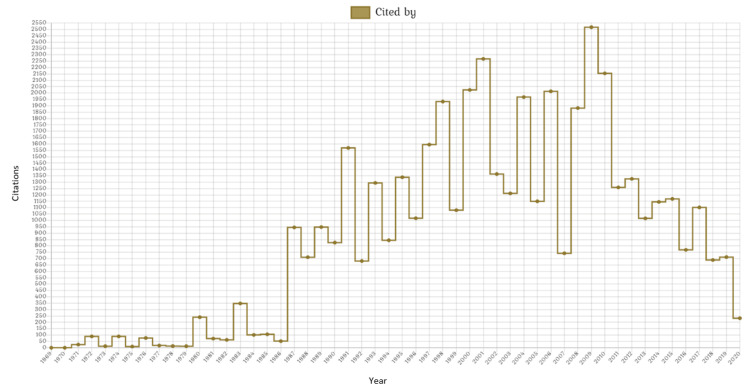
Article citations per year between 1969 - 2020 Of the 44,824 citations that have all articles, 27,557 belong to chemical methods, 13,084 to physical methods, and 4,183 to genetic models. The year 1987 was the boom year, with an increase that was maintained for almost all the following years, reaching its highest point in 2009, but after this, the citations started to decrease over the years. The growth wasn´t constant; it had ups and downs. The year 2007 with 742 citations was out of place, unlike the past year with 2013 citations.

All articles together have a total of 44,824 citations, that is, more than 34 citations on average per publication. In the chemical models, 819 of the 879 articles have already been cited at least once, for a total of 27,557 citations. In physical models, 288 of the 307 have citations, for a total of 13,084. In the genetic models, only nine of the 132 have not yet been cited out of the 4,183 citations they have. The distribution between years and the total of citations is analyzed in Figure 3.

**Figure 4:**
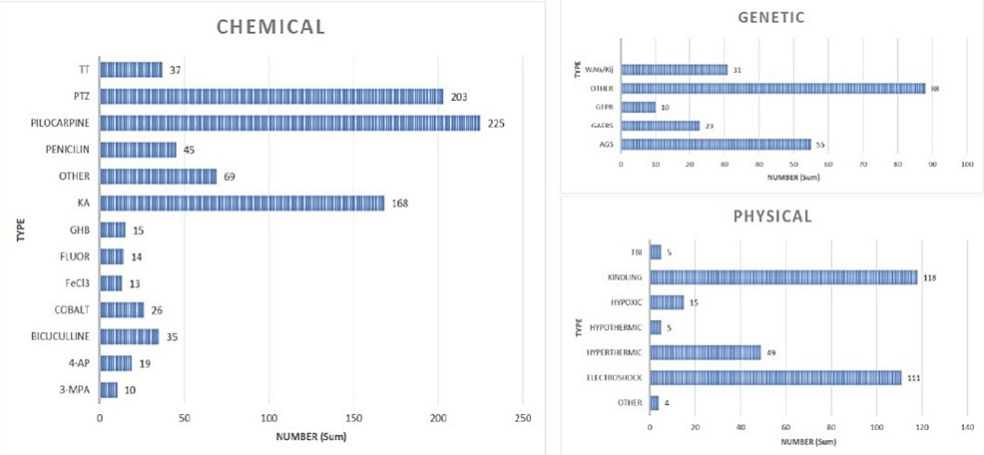
Number of articles according to experimental model A) Chemical models: in "pilocarpine," we group the pilocarpine and pilocarpine-lithium methods; in "penicillin," we also take into account the models induced by other beta-lactams. In "TT," we group toxins with mechanisms of action like tetanus toxin and picrotoxin. B) physical models: "kindling" refers only to electric kindling. C) Genetic models. TT: tetanus toxin; PTZ: pentylenetetrazol; KA: kainic acid; GHB: gamma hydroxy-butyrate; FeCl3: iron chloride; 4-AP: 4 aminopyridine; 3-MPA: 3 mercaptopropionic acid; WAG/Rij: Wistar Albino Glaxo Rijswijk (WAG/Rij) rats; GEPR: genetically epilepsy-prone rats; GAERS: genetic absence epilepsy rat from Strasbourg; AGS: audiogenic seizures; TBI: traumatic brain injury

The 1,318 articles used more than 50 different experimental models of epilepsy, of which the most used in these years were the models of epileptic seizures induced by pilocarpine (225), PTZ (203), KA (168), electric kindling (118), and electroshock (111). Models that were used in less than four experiments were grouped under "other". For graphical purposes, when a model was the result of some modification to the original method, it was grouped within the original model. The classified values are shown in Figure 4.

In terms of international impact, the most cited articles were “Decreased hippocampal inhibition and a selective loss of interneurons in experimental epilepsy” [12], “Apoptosis and proliferation of dentate gyrus neurons after single and intermittent limbic seizures” [13], and “Permanently altered hippocampal structure, excitability, and inhibition after experimental status epilepticus in the rat: the “dormant basket cell” hypothesis and its possible relevance to temporal lobe epilepsy” [14, 15]. They have 873, 686, and 603 citations, respectively. Table 1 shows the top 10 most cited articles.

**Table 1:** Top 10 most cited articles

First author	Title	Year	Journal	Cited by
Sloviter RS [12]	Decreased hippocampal inhibition and a selective loss of interneurons in experimental epilepsy	1987	Science	873
Bengzon J [13]	Apoptosis and proliferation of dentate gyrus neurons after single and intermittent limbic seizures	1997	Proceedings of the National Academy of Sciences of the USA	686
Sloviter RS [14]	Permanently altered hippocampal structure, excitability, and inhibition after experimental status epilepticus in the rat: the “dormant basket cell” hypothesis and its possible relevance to temporal lobe epilepsy	1991	Hippocampus	603
Mello LE [15]	Circuit mechanisms of seizures in the pilocarpine model of chronic epilepsy: cell loss and mossy fiber sprouting	1993	Epilepsia	568
Turski L [16]	Cholinergic mechanisms and epileptogenesis. The seizures induced by pilocarpine: a novel experimental model of intractable epilepsy	1989	Synapse	540
Ravizza T [17]	Innate and adaptive immunity during epileptogenesis and spontaneous seizures: evidence from experimental models and human temporal lobe epilepsy	2008	Neurobiology of Disease	501
Danober L [18]	Pathophysiological mechanisms of genetic absence epilepsy in the rat	1998	Progress in Neurobiology	454
Cossart R [19]	Dendritic but not somatic GABAergic inhibition is decreased in experimental epilepsy	2001	Nature Neuroscience	451
Liu DZ [20]	Brain and blood microRNA expression profiling of ischemic stroke, intracerebral hemorrhage, and kainite seizures	2010	Journal of Cerebral Blood Flow and Metabolism	422
Bernard C [21]	Acquired dendritic channelopathy in temporal lobe epilepsy	2004	Science	360

In addition, the journals that published the most articles were Epilepsia (99 articles), Epilepsy Research (86 articles), and Brain Research (53 articles). The top seven journals and their citations are shown in Table 2.

**Table 2:** Journals that published the maximum number of articles

Journal	Article	Citations
Epilepsia	99	3572
Epilepsy Research	86	3184
Brain Research	53	1756
Epilepsy and Behavior	42	828
Neuroscience	37	1563
Neuroscience Letters	36	771
Neurobiology of Disease	31	1785

Regarding the language of the articles, 1,240 articles (94.08%) are written in English and 33 (2.5%) in Chinese, while there are fewer than 10 articles originally written in French, Russian, Japanese, Persian, Turkish, Spanish, Ukrainian, German, Italian, Portuguese, Croatian, Polish, and Bosnian. All articles have been published in journals of 44 different countries; the top five are the USA (535 articles, 40.59%), the UK (244 articles, 18.51%), the Netherlands (194 articles, 14.71%), China (56 articles, 4.24%), and Ireland-Switzerland (45 articles, 3.41%).

Discussion

The study of epilepsy has had remarkable relevance in recent years, especially in the early 2000s. In fact, it should be noted that in the last 11 years of the time studied (2009-2020), the number of articles published has almost doubled. Productivity went from 0 articles in 1969 to three articles in 1979 and 14 articles in 1988. Sixty articles were produced in 2009, reaching the maximum in 2017 with 67 articles and having a fall in 2020, probably due to the COVID-19 pandemic. The citations, respectively, go as follows: 0, 13, 711, 2,517, 1,102, and 232. In other words, was expected that the citations would be reduced in 2010 because citations are influenced by the time that the articles were published as well as by the impact that they have. A uniform trend is followed in the choice of the experimental model, with a greater preference for chemical methods, followed by physical and limited participation in genetic methods. However, 1976, 1980, 1994, 2010, and 2011 were five exceptional years, because in 1976 and 1980, the decline in the use of chemical methods resulted in these being discreetly overtaken by physical methods, and in 1994, 2010, and 2011, there were more articles on genetic methods than on physical methods.

In recent times, the preference for chemical methods has become even more evident, and a near abandonment of genetic methods has been highlighted, which corresponds to the discovery of several new chemicals that can produce epileptic seizures. In addition to the ease of use and reproducibility, taken in 2017, there were 60 articles using chemical methods, unlike the genetic and physical methods, where there were five and 12 articles, respectively. While genetic models were expected to be the main choice in the early years, studies with other animals such as dogs, cats, or primates were excluded from this research, so the prevalence of genetic models actually declined significantly. On the other hand, in the beginning, the growth of chemical and physical methods was similar, until 1987, when chemical methods took off with nine articles, unlike physical methods with just four. After this point, the growth of the chemical methods has not stopped. The physical models are decreasing, but these articles have more international impact. Once published, we see that the physical models are the most cited, with an average of 42.61 citations per article, compared to 31.68 of the genetic models and 31.35 of the chemical ones.

Three chemical methods stand out against the others: pilocarpine, PTZ, and kainic acid. More particularly, pilocarpine is the most commonly used method in rats. The choice of this substance was expected due to its wide range of applications. With this model, rats start with automatisms, and after 90-180 minutes with high-voltage spikes, they give way to limbic motor seizures resulting in brain damage, which only happens with the highest dose but also increases the mortality rate. That is, the pilocarpine model is useful for studying acute seizures as well as the chronic effects that result from epilepsy. Due to the high rate of mortality, the new models introduce its use with other substances, like lithium, which leads to a decrease in mortality. It also shows that lithium without pilocarpine at any dose doesn´t cause seizures [22, 23]. Another model with high acceptance is electric kindling, which is mostly performed by stimulation in the brain amygdala of the rat after the placement of an electrode by stereotactic surgery. Among the reasons for its high diffusion, it is very useful to measure the progression of the convulsive state according to the stages described by Racine and also allows the exact measurement of electroencephalographic activity during and after the ictal period [24, 25]. It should be noted that the beginning of our study period coincides with Goddart's introduction of kindling in 1969 [24].

Temporal lobe epilepsy has a high resistance rate to antiepileptic drugs. It is a very impactful field within experimental models of epilepsy since the three most cited articles address this pathology and others within the top 10. This high scientific impact was also due to journals usually including experimental epilepsy as a fundamental part of their publications, such as Epilepsy, Epilepsy Research, Brain Research, and Epilepsy and Behavior, among others, which are usually a certainty of an article with high scientific impact. Unfortunately, the study of experimental epilepsy is almost non-existent in low-income countries, where the largest population with epilepsy is concentrated, and its collaboration with the United States of America is very low, which is the country with the highest productivity in epilepsy. In addition to economic barriers, another problem with this lack of collaboration is language, since less than 5.9% of publications on experimental epilepsy are published in a language other than English.

## Conclusions

Epilepsy is a common disease that warrants extensive research given its high refractoriness to treatment, with approximately one-third of cases proving resistant, and its disabling nature. Experimental models of epilepsy in rats have grown substantially in the last 10 years. Most researchers opt for experimental models using chemical induction of seizures with agents such as pilocarpine, PTZ, and KA for ease of use. However, electrical models such as kindling and electroshock have had more impact due to the higher number of citations for each of the papers and their publications in high-impact journals. More recently, genetic models of rat epilepsy have been less frequently used than previous methods. Articles on drug-resistant epilepsies are being published and cited more frequently. Although this shows that much has been done in what is currently known about epilepsy, there is much more to be investigated. This review highlights and acknowledges the contribution of rat models of epilepsy to our current understanding of epilepsy.
